# Genome-Wide Identification and Characterization of Olfactory Receptor Genes in Silver Sillago (*Sillago sihama*)

**DOI:** 10.3390/ani13071232

**Published:** 2023-04-01

**Authors:** Minghui Ye, Xinghua Lin, Yulei Zhang, Yang Huang, Guangli Li, Changxu Tian

**Affiliations:** 1 Fisheries College, Guangdong Ocean University, Zhanjiang 524088, China; ymh868860@163.com (M.Y.); zhangyl@gdou.edu.cn (Y.Z.);; 2 Guangdong Research Center on Reproductive Control and Breeding Technology of Indigenous Valuable Fish Species, Guangdong Provincial Engineering Laboratory for Mariculture Organism Breeding, Guangdong Provincial Key Laboratory of Aquatic Animal Disease Control and Healthy Culture, Zhanjiang 524088, China

**Keywords:** *Sillago sihama*, olfactory receptor genes, gene expansion, adaptive evolution

## Abstract

**Simple Summary:**

Silver sillago (*Sillago sihama*) is highly sensitive to environmental stimuli, which restrict the development of its breeding industry. Olfactory receptor (OR) genes play a critical role in the ecology of stress behavior in fishes. A total of 141 OR genes were identified in *S. sihama*, including 112 intact genes, 26 truncated genes, and three pseudogenes. The OR genes were classified into six groups, of which β, γ, δ, ε, and ζ groups belonged to type I; and η group belonged to type II. The type I OR genes contained almost all conserved motifs, while type II OR genes mainly retained conserved motifs 7(3), 1, 10, 4, and 2. OR genes were mostly distributed on LG 1, LG9, LG11, and LG12. Of all OR genes, 36.23% (50 genes) showed significant expansion in *S. sihama*. Ka/Ks analysis demonstrated that 227 sites were under purifying selection, while 12 sites were under positive selection, including eight genes in the OR2A12 gene subfamily. Sixty-one genes (44.20%) displayed differential expression under hypoxic stress. The OR gene expanded mechanisms revealed that positive selection promotes *S. sihama* efficient adaptation to environmental changes.

**Abstract:**

Olfactory receptor (OR) genes are essential in the specific recognition of diverse stimuli in fish. In this study, a total of 141 OR genes were identified in silver sillago (*Sillago sihama*), a marine fish sensitive to environmental stimuli, including 112 intact genes, 26 truncated genes, and three pseudogenes. A phylogenetic tree analysis elucidated that the OR genes of *S. sihama* were classified into six groups, of which β, γ, δ, ε, and ζ groups belonged to type I, and the η group belonged to type II. The type I OR genes contained almost all conserved motifs (*n* = 62), while type II OR genes mainly retained conserved motifs 7(3), 1, 10, 4, and 2 (*n* = 39). OR genes were mainly distributed on LG1, LG9, LG11, and LG12. Of all OR genes, 36.23% (50 genes) showed significant expansion in *S. sihama*. Ka/Ks analysis demonstrated that 227 sites were under purifying selection, while 12 sites were under positive selection, including eight genes in the OR2A12 gene subfamily. Sixty-one genes (44.20%) displayed differential expression under hypoxic stress. The identified OR genes explored the mechanism of environmental stress and ecological adaptation of *S. sihama*, and provided valuable genomic resources for further research on the olfaction of teleosts.

## 1. Introduction

Olfaction is an essential sensory modality in fish due to its action in feeding, migration, spawning, and predator avoidance [1,2]. Fish can obtain environmental information by sensing stimuli through olfaction, among which olfactory receptors (ORs) play an important role in recognizing stressors [3]. Fish recognize amino acids, steroids, prostaglandins, cholic acid, and other odorous molecules in the surrounding water environment through the OR and can detect changes in the environment [4]. With over 34,000 species, teleost fish have drastically changed morphology, physiology, behavior, ecology, and habitat, and are an ideal model to study the evolution of the olfactory system in aquatic environments [5]. Recent studies have found the emergence of new respiratory structures (swim bladders and lungs) in early teleost fish, possibly related to expansion of the OR gene family that was sensitive to air molecules [6]. Niimura et al. explored the influence of different environments and ecology on the evolution of expansion and contraction of OR gene families [7]. Expansion or contraction of teleost fish OR gene families indicates a mechanism of adaptation and evolution to environmental changes in teleost fish.

In vertebrates, odorant molecules are primarily detected by olfactory sensory neurons, and each expresses an OR [8]. Vertebrate chemosensory receptor genes were encoded by four large multigene families, olfactory receptor (OR), trace amine-associated receptor (TAAR), and vomeronasal receptor type 1 and 2 (V1R and V2R) [9]. Studies of the evolution of the vertebrate chemosensory receptor gene repertoire have focused mainly on mammalian OR genes. Far less research has been undertaken on the OR genes of teleost fish. The OR genes are G-protein-coupled receptors (GPCRs) in the class of rhodopsin, with seven α-helical transmembrane ™ conserved domains [10]. The OR gene family is one of the largest gene families in vertebrate genomes, with 1000 OR genes in mammals [11], while teleost fish possess only about 100 OR genes [12]. The number and proportion of functional OR genes reveal the importance of olfactory function to species and the adaptability of olfaction to environmental needs [13]. Although a limited number of teleost fish have been analyzed in previous studies, a wide range of variation and diversity has been observed [14,15]. There are only 67 OR genes in the sea lamprey (*Petromyzon marinus*), 176 in the zebrafish (*Danio rerio*), and 159 in the stickleback (*Gasterosteus aculeatus*) [7]. During evolution, the expansion of gene duplication and the loss of pseudogenes has led to large differences in the number of OR genes [2,16,17].

The types and expression patterns of OR genes vary among different species. OR genes in vertebrates are divided into two major types, such as type I (α, β, γ, δ, ε, and ζ) and type II (η, θ, κ, and λ) [15,18]. In mammals, α and γ groups are responsible for detecting airborne odors, and are excited by volatile odorant molecules in terrestrial environments [7]. Most teleost fish retain only six subfamilies (β, δ, ε, ζ, and η) to recognize water-soluble odorant molecules [19,20]. The β-groups can sense water-soluble and volatile odor molecules at the same time [15]. However, θ, κ, and λ groups are considered to be non-OR genes, because they are not to be expressed in the olfactory epithelium [19]. OR genes are expressed in the nasal cavity and widely expressed in other body parts, and play an important physiological role [21], indicating that OR genes in specific tissues probably have specific biological functions. With the advancement of high-throughput sequencing technology, the OR gene family has been identified in several fish species, including large yellow croaker (*Larimichthys crocea*) [18], Chinese perch (*Siniperca chuatsi*) [19], common carp (*Cyprinus carpio*) [22], and blunt snout bream (*Megalobrama amblycephala*) [20]. Identifying the OR gene family at the genome-wide level serves as a gene repository for the enrichment of ORs in vertebrates and provides a foundation for elucidating adaptive evolutionary mechanisms of environmental stress in fish.

Silver sillago (*Sillago sihama*), also known as smelt-whitings or sand borers, is a bottom-dwelling fish widely distributed on the coastal beaches of the Indo-West Pacific Ocean [23]. This fish has been widely cultured in China due to its high quality and nutritional value. *S. sihama* is sensitive to environmental stimuli and has the habit of drilling sand to avoid seine-net and other environmental hazards [24]. Lin et al. [25] reported the genome sequence of this species and found that OR genes were significantly expanded in the genome. In this study, the members of the OR gene family were identified and analyzed to unravel phylogenetic relationships, gene structures, conserved motifs, chromosomal locations, and gene duplications in the *S. sihama* genome. The results deepen the understanding of the origin and adaptive evolution of fish OR genes, and provide new ideas for exploring the genetic evolution patterns under stress conditions.

## 2. Materials and Methods

### 2.1. Ethics Statement

All experimental protocols were approved by the Animal Research and Ethics Committee of Guangdong Ocean University (201903003), Zhanjiang, China. The study does not include any endangered or protected species. The fish were anesthetized by immersion in eugenol before sampling the fish tissues.

### 2.2. Data Preparation

All available OR gene sequences of six teleost fish were downloaded from the National Center for Biotechnology Information (NCBI) (https://www.ncbi.nlm.nih.gov/ (accessed on 20 May 2022)) and Ensemble (http://asia.ensembl.org/ (accessed on 20 May 2022)), including large yellow croaker (*Larimichthys crocea*), tilapia (*Oreochromis niloticus*), zebrafish (*Danio rerio*), medaka (*Oryzias latipes*), stickleback (*Gasterosteus aculeatus*), and fugu (*Takifugu rubripes*) (Table S1). Genomic and genome annotation data of *S. sihama* were obtained from Lin et al. [25] (Table S2). The OR gene-coding sequences of *S. sihama* were translated to obtain amino acid sequences using the EditSeq program of DNASTAR Lasergene 7. The genome annotation data of *L. crocea* were obtained from Ensemble. The protein sequences of the *S.sihama* and 10 other teleosts were obtained from NCBI, including *L. crocea* (GCA_003845795.1), *T. rubripes* (GCA_000180615.2), *G. aculeatus* (GCA_006229165.1), *O. latipes*(GCA_004347445.1), *D. rerio*(GCA_000002035.4), *O. niloticus* (GCA_001858045.3), West Indian Ocean coelacanth (*Latimeria chalumnae*) (GCF_000225785.1), Chinese sillago (*Sillago sinica*) (http://dx.doi.org/10.5524/100490 (accessed on 13 January 2021)), spotted gar (*Lepisosteus oculatus*) (GCA_000242695.1), and Southern platyfish (*Xiphophorus maculatus*) (GCA_002775205.2).

### 2.3. Gene Identification

A total of 542 OR gene protein sequences from *L. crocea* (*n* = 111), *O. niloticus* (*n* = 266), and *D. rerio* (*n* = 165) were used as query sequences to conduct a TBLASTN search (Version: 2.7.1+) in the *S. sihama* genome, with an e-value cut-off of 1 × 10^−5^, and the predictive limit of intron size at default parameters [25]. Fasta Extract functions in the TBtools (v1.098696) software were used to extract these gene regions from the genome [25]. Then, the obtained OR genes were annotated based on the existing *S. sihama* genome annotation results. To further verify the accuracy of the annotations, the obtained candidate OR genes were compared to the NCBI non-redundant database (BLASTX), and the corresponding non-OR GPCRs were discarded. The genes with complete open reading frames (ORF) and transcriptome alignment results were defined as OR genes. Finally, the candidate OR genes were divided into three categories: “intact genes” were at least 250 amino acids with no interrupting stop codons or frameshifts, and could code for 7 TM domains; “pseudogenes” with frameshifts or stop codons; and “truncated genes” without start or stop codons, but well matched to the known OR genes [19]. OR gene sequences were submitted to ExPASy (https://web.expasy.org/protparam/ (accessed on 28 May 2022)) to predict the number of amino acids, protein molecular weight, isoelectric points (pI), and instability index [26]. The conserved domains of *S. sihama* OR were predicted by SMART (http://smart.embl-heidelberg.de/ (accessed on 28 May 2022)) [27]. The exon/intron structures of *S. sihama* OR genes were displayed by Gsds 2.0 (http://gsds.cbi.pku.edu.cn/ (accessed on 30 May 2022)). Only intact and truncated genes were analyzed, as pseudogenes were not transcribed.

### 2.4. Phylogenetic Analysis and Classification

A phylogenetic tree was conducted based on the OR gene sequences of *S. sihama*, and the known functional OR gene sequences of six teleost fish, including *L. crocea*, *O. niloticus*, *D. rerio*, *O. latipes*, *G. aculeatus*, and *T. rubripes*. The amino acid sequences of OR genes were aligned using the Clustal W program in MEGA-X, with default parameters. Multiple-sequence alignment results and tree information were obtained. The neighbor-joining (NJ) phylogenetic tree was built using MEGA-X with the following parameters: bootstrap method and 1000 bootstrap replications [28,29]. Each branch was assigned according to the known zebrafish OR [15]. According to the classification of zebrafish OR genes, OR genes were classified into putative subfamilies using BLASTP [7]. The phylogenetic tree was visualized using Evoview (https://www.evolgenius.info/evolview/#login (accessed on 10 July 2022)) [30].

### 2.5. Chromosomal Distribution and Motif Analysis

The chromosomal location and structure information of OR genes were obtained from the genome annotation data [25]. The OR genes were mapped to *S. sihama* chromosomes, which were visualized by the Mapchart software [31]. Pseudogenes and OR genes located in scaffolds were not displayed. The conserved motifs of OR genes were analyzed using the MEME Suite (version 5.1.1, http://meme-suite.org/tools/meme (accessed on 27 March 2023)), with a maximum of 10 motifs. The conserved motifs were visualized by the TBtools software (version 0.665).

### 2.6. Collinearity Analysis

In order to determine the degree of homology of OR genes between the genomes and the evolutionary characteristics of OR genes, *L. crocea*, which was closely related to the evolution of *S. sihama* OR genes, was selected as a collinear species. The *S. sihama* and *L. crocea* protein sequences were analyzed through “all-against-all” alignment [32]. For inter-species collinearity analysis, the alignment results and the gene-structure annotation file were subjected to MCScanX [33]. The *S. sihama* and *L. crocea* OR gene homologous gene pairs were highlighted, and evolutionary relationships were determined. The collinearity relationships were displayed using the TBtools software (version 0.665) [34].

### 2.7. Adaptive Evolution Analysis of S. sihama OR Genes

The expansion and contraction gene families among *S. sihama* and 10 other teleost fish (*L. crocea*, *T. rubripes*, *G. aculeatus*, *O. latipes*, *D. rerio*, *O. niloticus*, *L. chalumnae*, *S. sinica*, *L. oculatus*, and *X. maculatus*) were identified by CAFE [35]. The number of gene families of each ancestor was estimated by the birth mortality model, predicting the number of gene family expansion and contraction gene families. The expanded data of *S. sihama* OR genes were obtained.

The OR gene CDS sequences of *S. sihama* were established by BLAST. The CDS sequences were screened from BLASTN, with an e-value cut-off of 1 × 10^−20^, namely, “all vs. all” alignment. The gene pairs that were more than 75% similar were screened. The sequence length of the alignment results was then obtained using the Samtools tool. The criteria for identifying gene duplications are as follows: the length of the alignable sequence covers 75% (relative to longer genes), and the similarity of aligned regions is 75% [36]. The duplicated gene pairs were obtained using the Clustal W codon alignment tool. Finally, the selective pressure was estimated using the CDS sequences of duplicated gene pairs [37]. The Ks/Ks values (synonymous and non-synonymous substitution rates) of the duplicated gene pairs were calculated using the Simple Ka/Ks Calculator (NG) program of the TBtools software (v1.098696).

### 2.8. Expression Pattern of OR Genes under Hypoxic Stress

*S. sihama* was sensitive to hypoxic stress. This study verified the expression patterns of the OR gene family in *S. sihama* under hypoxic stress in the gill, heart and liver tissues. The hypoxia transcription sequencing databases (RNA-seq) of *S. sihama* were obtained from Saetan et al. [38,39] and Tian et al. [40]. Gene expression level under hypoxic stress was calculated by the FPKM (fragments per kilobase of transcript per million fragments mapped) method. The expression pattern of the OR genes under hypoxia stress was drawn using the TBtools (v1.098696) software.

## 3. Results

### 3.1. Identification of the S. sihama OR Gene Family

A total of 141 OR genes were identified in the *S. sihama* genome, including 112 intact genes (putatively functional genes), 26 truncated genes, and three pseudogenes (Table 1). The length of the encoded amino acid sequences of OR genes ranged from 158 (*OR10C1*) to 384 (*OR8D4*) a.a., with an average of 315 a.a. The molecular weight ranged from 17.99565 (*OR10C1*) to 54.7859 (*OR10AG1.2*) kDa. The pI value ranged from 8.14 (*OR8D4*) to 10.08 (*OR4N5*) (Tables S2 and S3). The 110 OR genes had a single-exon structure, and 28 OR genes (20%) consisted of 2–5 coding exons (Figure S1).

### 3.2. Classification and Phylogenetic Analysis of the OR Gene Family

**Figure 1:**
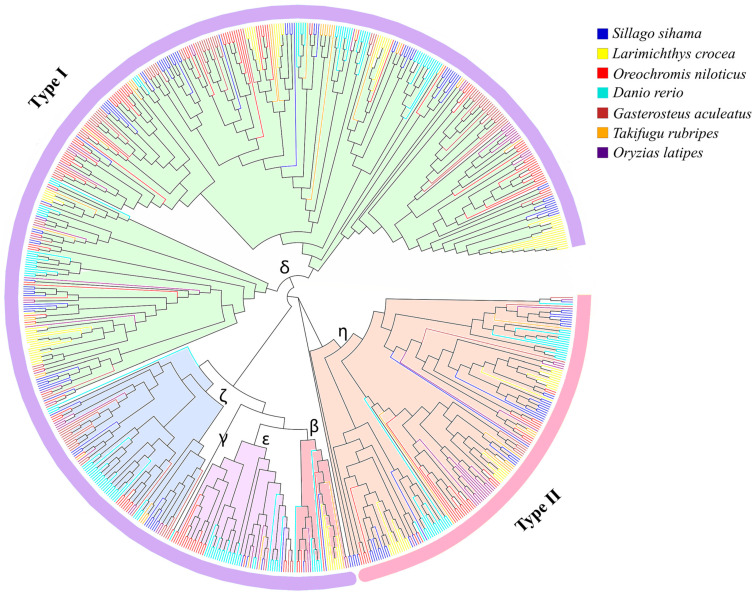
Phylogenetic trees of OR genes from *S. sihama* and six teleost fish functional genomes (*n* = 745). Phylogenetic trees were constructed using amino acid sequences from seven species (bootstrap copy number: 1000). The phylogenetic tree is divided into six groups (β, γ, δ, ζ, ε, and η). Groups named β to η are signed on the branches. The type I genes are surrounded by the purple line, and the type II genes are surrounded by the pink line.

A phylogenetic tree was constructed with 138 OR genes (intact genes and truncated genes) of *S. sihama* and 607 OR functional genes of six teleost fish (Figure S2). A phylogenetic tree of OR genes showed that OR genes of *S. sihama* were consistent with the evolutionary relationship of selected species, and had a closer relationship with *L. crocea* and *O. niloticus* (Figure 1). The number of OR genes was variable among teleosts (Table 1). Of the OR genes of *S. sihama*, 138 were classified into five groups, of which δ (*n* = 78), ζ (*n* = 10), ε (*n* = 4), and β (*n* = 2) groups belonged to type I, and η (*n* = 43) group belonged to type II (Figure 1, Table 1).

### 3.3. Conserved Motifs and Chromosomal Distribution of S. sihama OR Genes

**Figure 2:**
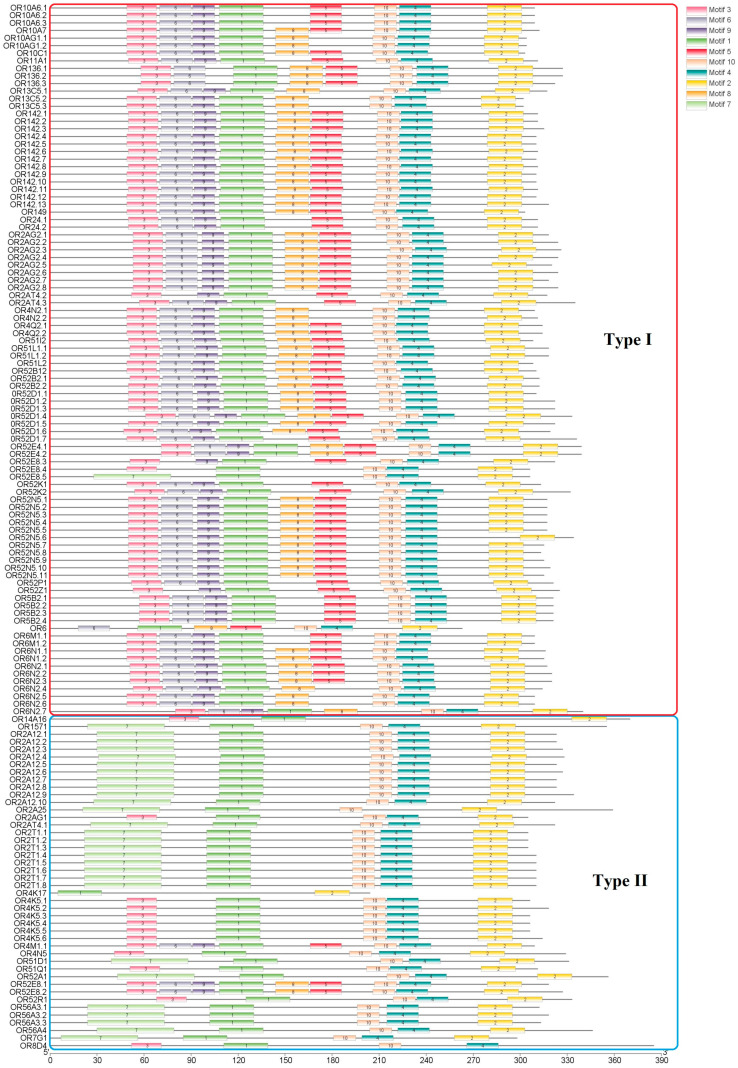
Conserved motifs of OR genes in *S. sihama*. Different colors differentiate 10 different motifs. The order of the motifs was 7, 3, 6, 9, 1, 8, 5, 10, 4, and 2. The horizontal axis shows the number of amino acids from the N-terminal to the C-terminal. The OR gene conserved motifs in the red border region belong to type I, while those in the blue border region belong to type II.

**Figure 3:**
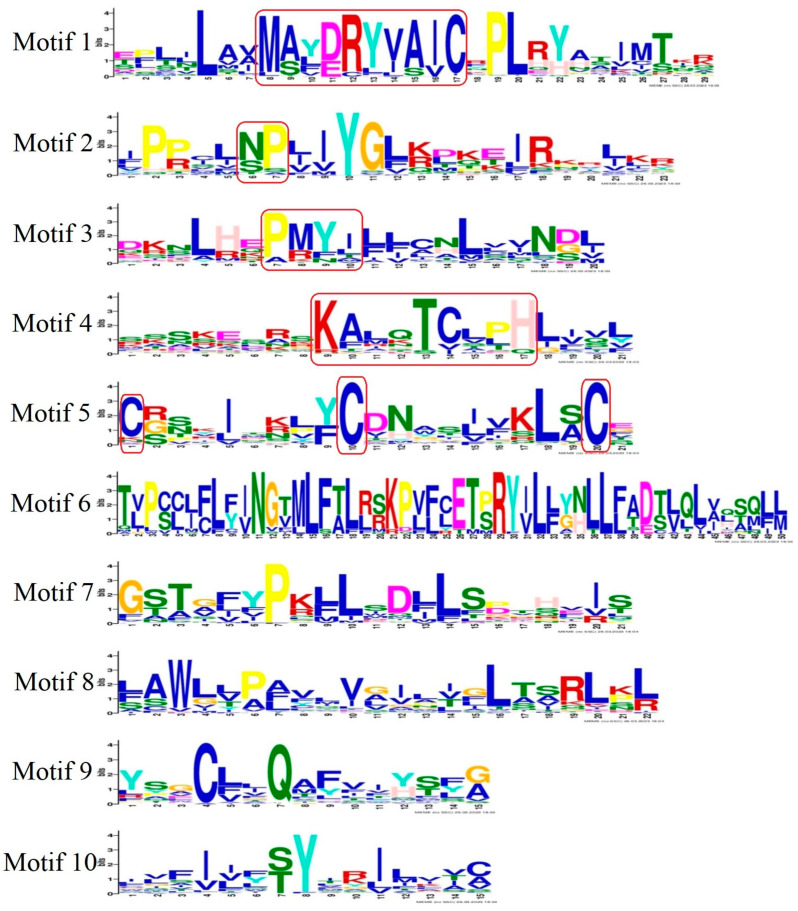
Logo representation of the 10 best-conserved motifs in OR genes. The height of the amino acid code represents the conserved degree of the motifs. The height of the letters in the logo indicates the relative frequency of amino acid occurrence, and is proportional to the level of sequence conservation. The sequence outlined by red rectangles was a functionally conserved motif in motifs 1, 2, 3, 4, and 5.

**Figure 4:**
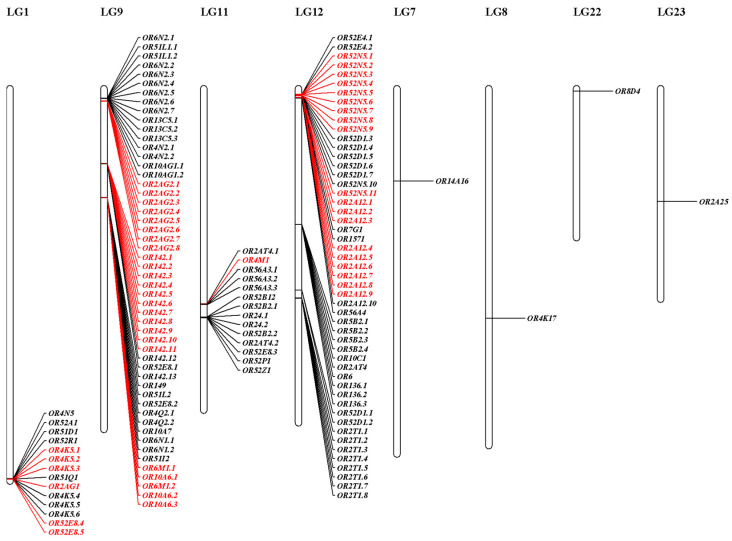
Chromosomal distribution of *S. sihama* OR genes. The majority of *S. sihama* OR genes were organized in seven clusters on four chromosomes. The other four OR genes were located on four chromosomes. The genes highlighted in red are expanded genes.

The ten conserved motifs were examined from OR proteins of *S. sihama* (Figure 2 and Figure 3). The OR gene subfamily shared a similar motif composition. The type I OR genes contained almost all conserved motifs (*n* = 62), while type II OR genes mainly retained conserved motifs 7(3), 1, 10, 4, and 2 (*n* = 39). Chromosomal distribution of OR genes in *S. sihama* showed that the OR genes were mapped on LG1 (*n* = 14), LG9 (*n* = 52), LG11 (*n* = 14), LG12 (*n* = 51), LG7 (*n* = 1), LG8 (*n* = 1), LG22 (*n* = 1), and LG23 (*n* = 1). The remaining three OR genes were located on the unmapped scaffolds (Figure 4). There are three OR gene clusters on LG9 and LG12, and one OR gene cluster each on LG11. The OR genes in LG9 and LG12 were significantly expanded (Figure 4).

### 3.4. Collinearity Analysis of S. sihama OR Genes

**Figure 5:**
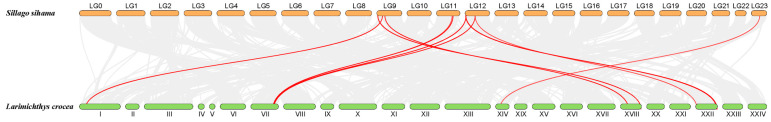
Collinearity relationship of OR genes in *S. sihama* and *L. crocea.* Orange bars represent the *S. sihama* chromosome. Green bars represent the *L. crocea* chromosome. The grey lines represent the collinearity relationship between *S. sihama* and *L. crocea* genes. The red lines represent the collinearity relationship between *S. sihama* and *L. crocea* OR genes.

Collinearity analysis of *S. sihama* and *L. crocea* revealed 22 homologous gene pairs in the OR genes (Figure 5; Table S4). The OR genes of *S. sihama* located on LG9, LG11, LG12, and LG23 were homologous to *L. crocea.* LG9 corresponds to I and XVIII, LG11 corresponds to VII, LG12 corresponds to VII and XXII, and LG23 corresponds to XIV (Table S4). Of these, the *OR6N2.4* and *OR2AG.4* in LG9 were collinear with XVIII. *OR2AT4.2* in LG11 and *OR2AT4.3* in LG12 were collinear with VII. *OR2A12.1* was collinear with VII and XXII (Table S4). These results suggested that *S. sihama* OR genes underwent chromosome rearrangement during evolution.

### 3.5. Adaptive Evolution Analysis of S. sihama OR Genes

**Figure 6:**
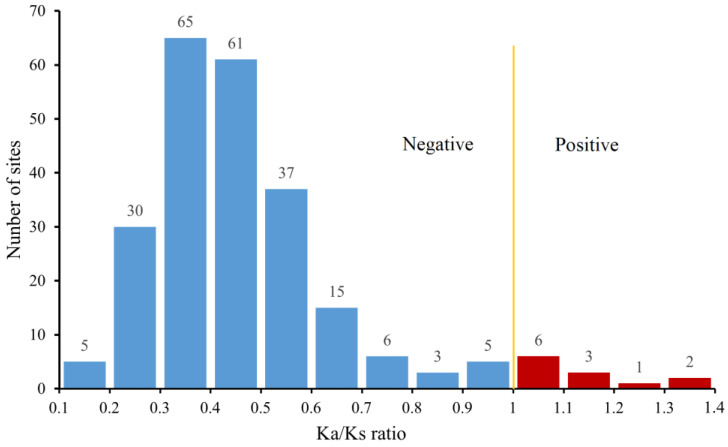
Selection pressure imposed on the OR gene pairs. The X-axis represents the range of the Ka/Ks ratio. The Y-axis represents the number of selection sites detected.

A total of 36.23% (50/138) genes were expanded in *S. sihama* (Table S2). Of these, the expanded genes mainly occurred in the three subfamilies of the δ group, including OR142 (*n* = 11), OR52N5 (*n* = 10), and OR2AG2 (*n* = 8). In addition, the OR2A12 gene subfamily in the η group was expanded (*n* = 9). Selection pressure analysis of OR duplicated genes showed that *S. sihama* OR genes were detected in 239 sites (gene pairs), of which the Ka/Ks ratios of 12 sites (5%) were higher than 1.0 (under positive selection); the Ka/Ks ratios of 227 sites (95%) were below the value of 1.0 (under purifying selection); and 209 sites (87.4%) had Ka/Ks ratios between 0.2 and 0.6 (Figure 6). The 12 gene pairs of the OR2A12 gene subfamily (eight genes) were positive selections belonging to the η groups and located on LG12. In addition, the global ratios of Ka/Ks were below 1.0 for OR genes in *S. sihama* (Table S5).

### 3.6. Gene Expression Patterns of S. sihama OR Genes under Hypoxic Stress

**Figure 7:**
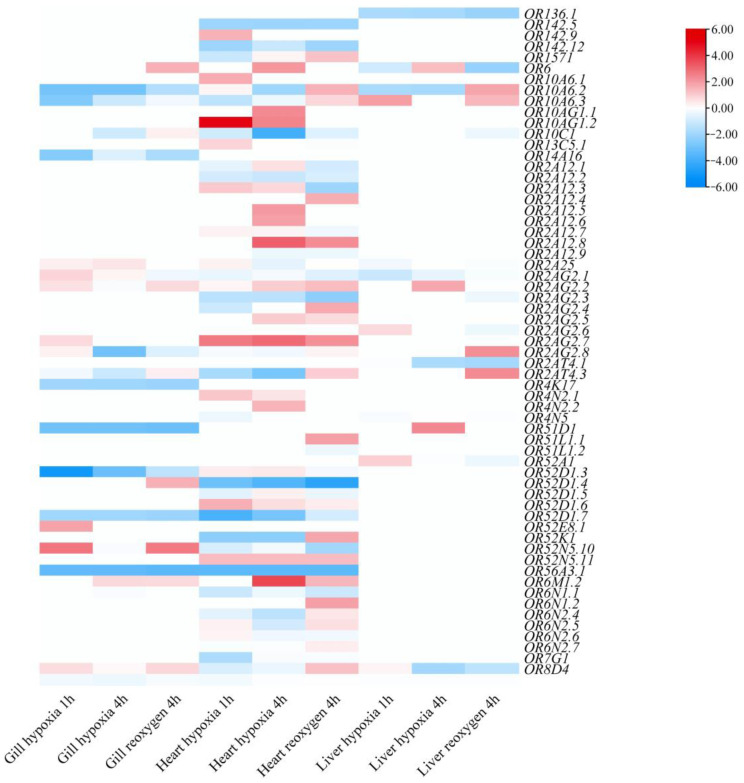
Heatmap of differential expression profile of *S. sihama* OR genes under different levels of hypoxia related to normal conditions for the gill, heart, and liver. Lighter to darker shades of red and blue indicate increasing upward and decreasing downward regulation of gene expression, respectively. White squares indicate absence of up- or down-regulation.

A total of 61 OR genes (44.20%) displayed differential expression under hypoxic stress (Figure 7; Table S6). Among them, 16 genes in gill tissue, 35 genes in heart tissue, and nine genes in the liver tissue were differentially expressed in the three treatment groups. Notably, the expression levels of expanded OR genes were increased during hypoxic stress, such as *OR2A12.8*, *OR2AG2.7,* and *OR6M1.2*. In the gill tissue, the expression of *OR52N5.10* was significantly up-regulated after 1 h of hypoxia and 4 h of re-oxygenation, and the expression of *OR52D1.3* was significantly down-regulated after hypoxic stress. In the heart tissue, the expression of *OR10AG1.2* was significantly up-regulated after 1 h of hypoxia, and the expression levels of *OR52D1.4* and *OR56A3.1* were significantly down-regulated after hypoxic stress. In the liver tissue, the expression of *OR10A6.2* was down-regulated after 1 h and 4h of hypoxia, and up-regulated after 4h of re-oxygenation. The results showed that the expression of *S. sihama* OR genes were significantly different under hypoxic stress (Table S6).

## 4. Discussion

### 4.1. Characterization of OR Genes in S. sihama

A total of 141 OR genes in the *S. sihama* genome were identified in this study. The average length of *S. sihama* OR genes was 315 amino acids, which was similar to a previous study in teleost [7]. The number of OR genes varied greatly in vertebrates, and the higher number indicated a heavier reliance on olfaction [19]. Terrestrial animals have a large number of OR genes adapted for their precise recognition of complex odor molecules in the air, while fish have a smaller number of OR genes that may indicate a response to an environment closer to the organism [42]. In mammals, OR genes are more abundant in adapting their sensitive olfactory function. For instance, mice (*Mus musculus*) encoded 1037, and humans (*Homo sapiens*) encoded 388 functional OR genes, indicating that mammals may evolve more OR genes to adapt to the terrestrial environment [43]. In teleosts, the number of OR genes is highly variable due to the complex and diverse ecological niches of each species [2,44]. The number of functional genes ranged from 20 in common mola (*Mola mola*), to 279 in barramundi fish (*Lates calcarifer*) [20]. *S. sihama* has the propensity to drill sand and is sensitive to environmental stimuli. In this study, 141 OR genes have been identified in *S. sihama*, and the number of OR genes is more than that of most known teleosts, indicating that the ORs of *S. sihama* play an important role in sensing the adaptive changes in the external environment.

The conserved motifs of OR genes have been identified in vertebrates [45], which mainly included the motif NX[TS]X in motif 2, the motif MA[FY][DE]RYVAIC in motif 1, conserved cysteine residue site in motif 5, the motif PMLNPFIY in motif 3, and the motif KAFSTCXSH in motif 4. Highly conserved regions and amino acid locations of OR genes were identified. Motifs 1, 2, 3, 4, and 5 of OR genes in *S. sihama* were highly conserved motifs (Figure 4), and the sequence and location were very similar to those of known teleosts and mammals [41,46]. Functionally important regions of proteins are identified by the conserved motif. However, the important regions of the specific protein function will not be conserved when members of OR genes have diverged functionally [47]. Interestingly, motif 5 was highly varied in *S. sihama* OR genes, which was considered a potential position for multifarious binding compounds to better accommodate thousands of odor molecules [19]. The OR gene structure of *S. sihama* consisted of different conserved elements. The OR genes of type I contained almost all conserved motifs. In contrast, type II mainly retained conserved motifs 7(3), 1, 10, 4, and 2. The type, order, and number of motifs in the same subfamily proteins of the *S. sihama* OR gene family were similar, but different from other subfamily proteins, indicating that the *S. sihama* OR gene family was stable at the molecular level in the process of evolution. Although the TBLASTN algorithm (word length) is limited in identifying small coding regions, which may miss genes composed of a large number of exons [48], we identified 28 (20%) multiple exon OR genes by TBLASTN. In the cichlid genomes, 9% of the OR genes with several coding exons were also found by TBLASTN [41], indicating TBLASTN is an effective method to identify OR gene families of *S. sihama*. Of course, more methods need to be tried in different species in the future to identify possible missing OR genes in fish. Based on OR gene structure of *S. sihama*, the 110 OR genes were single-exon structures, whereas nearly 20% of the OR genes were composed of several coding exons (Figure S1). In the study of cichlid genomes, multi-coding exon genes encoded the OR gene family [41]. For instance, the medaka, stickleback, and zebrafish OR genes can be encoded by multi-coding exon genes. Further functional gene studies must prove that multi-coding exon OR genes are active.

On a chromosome, two genes are arranged in a cluster when the distance is smaller than 1 Mb [43]. Previous studies have demonstrated that OR genes were arranged tightly into clusters in teleost fish. In zebrafish and Chinese perch, OR genes were arranged into seven major clusters on four chromosomes [19,46]. In channel catfish (*Ictalurus punctatus*)*,* OR genes were arranged into four major clusters [49]. In this study, seven major clusters on four chromosomes in the *S. sihama* OR genes were found. In the process of OR gene duplication, only tandem repeats were identified on the same chromosome, and no separation of related gene clusters was found. These gene clusters make the members of each OR gene family closely arranged in clusters into tandem repeating units [46], which suggests that the OR gene family may be built after complex duplication of gene clusters.

### 4.2. Phylogenetic Analysis of OR Genes in S. sihama

Consistent with the evolutionary characteristics of OR genes in teleosts, five groups (β, δ, ε, ζ, and η) of OR genes were identified in the genome of *S. sihama* (Figure 1). The phylogenetic relationships of OR genes among teleosts were consistent with their evolutionary relationships, similar to previous findings [25,28,49]. The OR genes originated early in chordate evolution, and the type I and type II genes diverged before the divergence of jawless and jawed vertebrates [18].

In this study, members of the group β were found in *S. sihama* and six species of teleosts, which were a few copies in fish (Table 1). Groups α and γ of type I have been absent in teleosts, but expanded in tetrapods. Although terrestrial animals have more OR genes than aquatic animals, aquatic animals retain diverse OR genes type [50]. Fish need more functionally differentiated OR genes to identify odor molecules, to adapt to the aquatic ecological environment [51]. The *S. sihama* OR genes mainly retained β, δ, ε, ζ, and η groups, which maintained the function of fish to recognize water-soluble odor molecules, consistent with the characteristics of the OR genes in fish [20]. Mammals require a large number of OR genes to adapt to the terrestrial ecological environment. Therefore, the numbers of δ, ε, ζ, and η groups were completely lost in mammals, whilst α and γ groups were significantly expanded. Amphibians retained nearly all OR genes groups except ζ, which are sensitive to water-soluble and airborne odorants and can adapt to their aquatic and terrestrial lifecycles [15]. However, the members of different groups may recognize different odors, while the same groups may recognize more subtle differences between similar odors, which may be the reason for the diversification of the teleost fish OR genes [52]. A recent study using comparative evolution to analyze the expansion of OR genes in aquatic species revealed a significant expansion of groups β and ε in freshwater fish, reflecting ecological adaptation [20]. The number of OR genes in each subfamily may represent the importance of a particular subfamily to the species, because subfamilies of OR genes that are important for the survival of the species are likely to expand in the genome through evolution [18]. Combined with the analysis of expanded OR genes in *S. sihama*, δ and η groups were significantly expanded, which may play an important role in adapting olfactory perception (Table 1).

### 4.3. Adaptive Expansion of OR Genes in S. sihama

The expansion and contraction of the OR gene repertoire in a lineage were controlled by a birth-and-death process. The adaptive evolution of olfaction involves recurrent gene duplications and losses [7]. Lin et al. [25] found that *S. sihama* had 100 expanded and 25 contracted gene families, through the expansion and contraction analysis of teleost fish using the birth mortality model. The OR genes of *S. sihama* were significantly expanded (50/138) during evolution by the birth mortality model analysis. The adaptive evolution of the gene families may be related to the evolution of phenotypic diversity and environmental adaptation. Increases in the gene number likely create additional targets of opportunity for beneficial mutations, and enhance the efficacy of positive selection [53]. In this study, the expression of 61 OR genes (44.20%) in *S. sihama* were significantly different in the heart, gill, and liver under hypoxic stress, indicating that environmental stress significantly affected OR gene expression. The heart is an important organ for fish to sense environmental changes in life activities. The expression of OR genes in heart tissue was significantly different under hypoxia stress, which may play an important role in protecting the heart from hypoxia damage [54]. We suspected that the OR genes in heart tissue may be more affected by environmental stress. For example, the expression of OR2A12 gene subfamily was significantly different under hypoxia stress in heart tissue, and the gene clusters formed by OR2A12 have positive selection. Environmental stress induces a stress response in fish, which directly affects fish behavior, physiology, and development [55]. Changes in the ecological environment may have selective pressure on the evolution of OR genes in *S. sihama*. Genes that affect the behavioral characteristics of species can expand in adaptive evolution, especially OR genes playing important functions in organism development, reproduction, and foraging [53]. Previous studies on rat OR genes have found that genes influencing behavioral traits can be subject to adaptive evolution [47]. Gene expansion of the OR may reduce the range of odorant molecules that bind to each receptor and output more precise olfactory signals [43]. Therefore, the evolutionary pressure of duplicate genes was evaluated, and multiple sites of *S. sihama* OR genes under negative selection pressure were identified (Figure 6), which maintained the relative stability of olfactory structure and function [19,49]. Nevertheless, the OR2A12 gene cluster at LG12 was significantly under positive selection pressure, leading to the expansion of OR genes during the adaptive evolution of *S. sihama*. Changes in selection pressure impact the genes encoding functional proteins, which is one of the reasons for the diversification of functions in the gene family [16]. This expansion mechanism provides the OR genes with the ability to adapt to changes in environmental conditions.

Gene expansion caused by gene tandem duplication promotes the formation of gene families, which have different, but related functions in adaptive evolution [13]. In this study, OR gene subfamilies were contiguous in gene clusters, which suggests that tandem duplication is one of the mechanisms for gene expansion [19]. In addition, OR genes belonging to the same clade were located in the same genome cluster in the phylogenetic tree, which is consistent with a previous study [56]. The OR genes in δ and η groups were significantly expanded (Table 1), especially in the η groups, which has positive selection pressure on the expanded genes. The expansion of *S. sihama* OR genes is due to tandem repeats and chromosomal rearrangements, which evolved rapidly through duplication and adaptive mutations [57]. *S. sihama* has the propensity to drill sand when frightened and immediately bury in the sand to avoid enemy harm. Olfactory receptor plays an important role in sensing environmental odor molecules; therefore, the sand-drilling habits of *S. sihama* may help combat environmental stimuli. Due to natural selection pressure, gene tandem duplication, and chromosome rearrangement, OR gene expansion reveals that it plays an essential role in its adaptive evolution process.

## 5. Conclusions

In this study, the OR genes were identified in *S. sihama*, a marine fish sensitive to environmental stimuli. A total of 141 OR genes were identified, including 112 intact genes, 26 truncated genes, and three pseudogenes. Of all OR genes, 36.23% (50 genes) showed significant expansion in *S. sihama*. The expanded genes occurred mainly in LG9 and LG12 gene clusters and were under positive selection pressure. The gene-expansion mechanisms revealed that positive selection promoted *S. sihama* efficient adaptation to environmental changes. Sixty-one genes (44.20%) displayed differential expression under hypoxic stress. Therefore, this study provides important information for analyzing OR genes and their expression.

## Figures and Tables

**Table 1:** Summary of the number of OR genes in different species.

Species	β	γ	δ	ζ	ε	η	Non-OR	Functional Genes	Truncated Genes	Pseudo Genes	References
*S. sihama*	2	0	78 (35)	10	4 (1)	43 (14)	1	112	26	3	-
*D. rerio*	6	1	54	40	13	28	1	143	1	21	Niimura [15]
*O. latipes*	3	0	33	9	3	19	1	68	6	24	Niimura [15]
*G. aculeatus*	1	0	71	18	4	7	1	102	5	52	Niimura [15]
*T. rubripes*	1	0	30	4	2	10	0	47	39	39	Niimura [15]
*O. niloticus*	5	1	91	27	3	31	0	158	101	7	Azzouzi et al. [41]
*L. crocea*	7	0	55	0	0	27	0	89	22	N/A	Zhou et al. [18]

Note: Numbers in parentheses indicate the number of expanded ORs in this group. Non-ORs include θ, κ, and λ groups.

## Data Availability

All data generated or analyzed in this study are reported in this published article. The genome annotation data of *S. sihama* in used for this study are accessible at the National Genomics Data Center (https://bigd.big.ac.cn/gwh) accession number GWHAOSB00000000, and the genomic accessible in NCBI under bioproject number PRJNA642704.

## Supplementary Materials

The following supporting information can be downloaded at: https://www.mdpi.com/article/10.3390/ani13071232/s1, Figure S1: The exon/intron structures of *S. sihama* OR genes; Figure S2: Phylogenetic trees of OR genes from *S. sihama* and six teleost functional genomes; Table S1: The protein sequence information of the OR functional gene of *Sillago sihama*, *Larimichthys crocea*, *Oreochromis niloticus*, *Danio rerio*, *Oryzias latipes*, *Gasterosteus aculeatus*, and *Takifugu rubripes*; Table S2: The chromosome location, amino acid and CDS sequences of *S.sihama*; Table S3: The OR genes and their basic information identified in *S.sihama*; Table S4: Twenty-two homologous gene pairs of the OR genes between *S. sihama* and *L. crocea*; Table S5: Selection pressure analysis of the OR duplicated gene pairs; Table S6: The expression patterns of *S. sihama* OR genes under hypoxic stress in the gill, heart and liver tissues.
