# Supplementary figures and images for: Genome-Wide Identification and Characterization of Olfactory Receptor Genes in Silver Sillago (Sillago sihama)
Source: Animals (Basel). 2023 Apr 1;13(7):1232. doi: 10.3390/ani13071232 (PMC10093537; doi:10.3390/ani13071232)

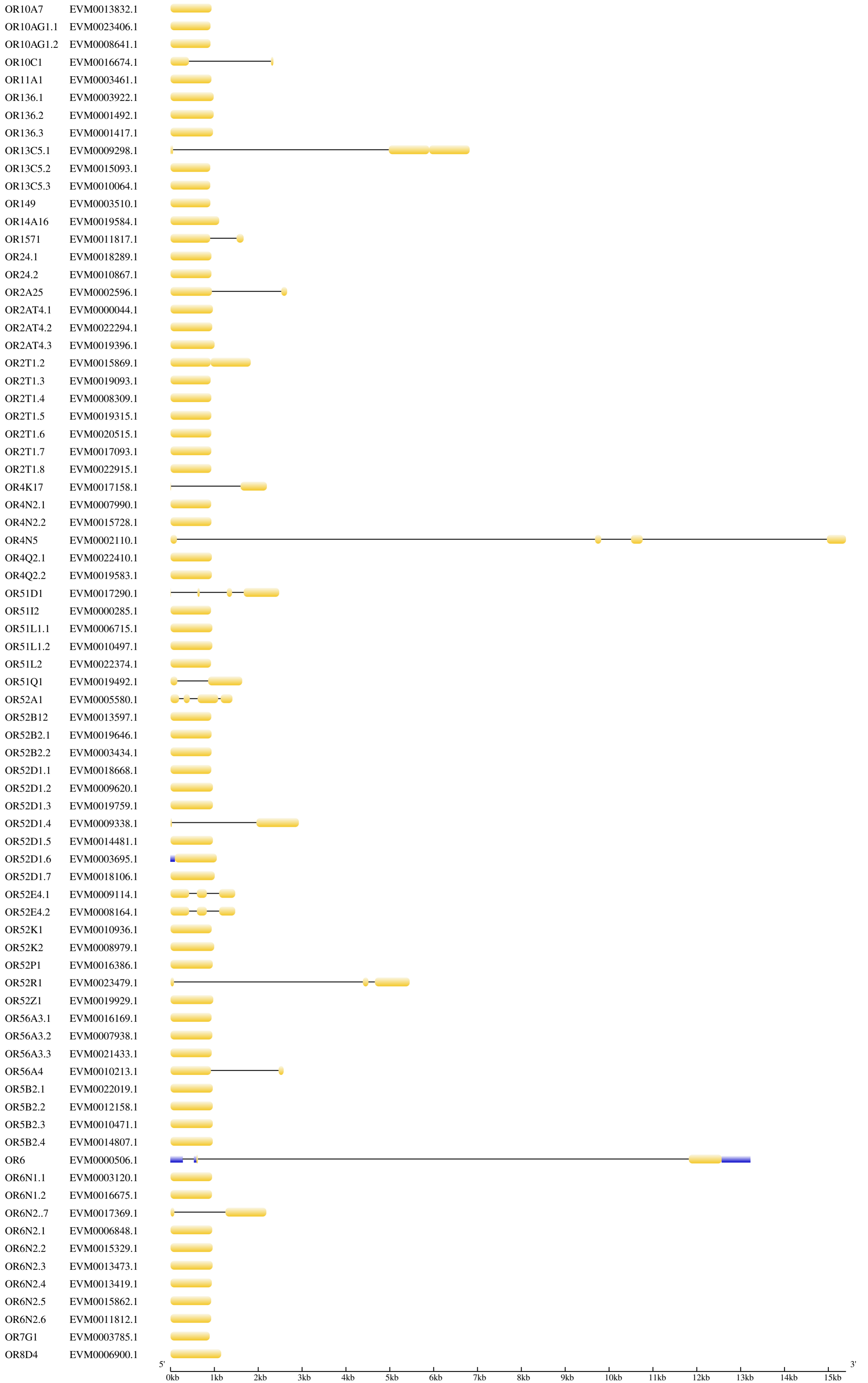

Legend:  
 CDS    UTR    Intron

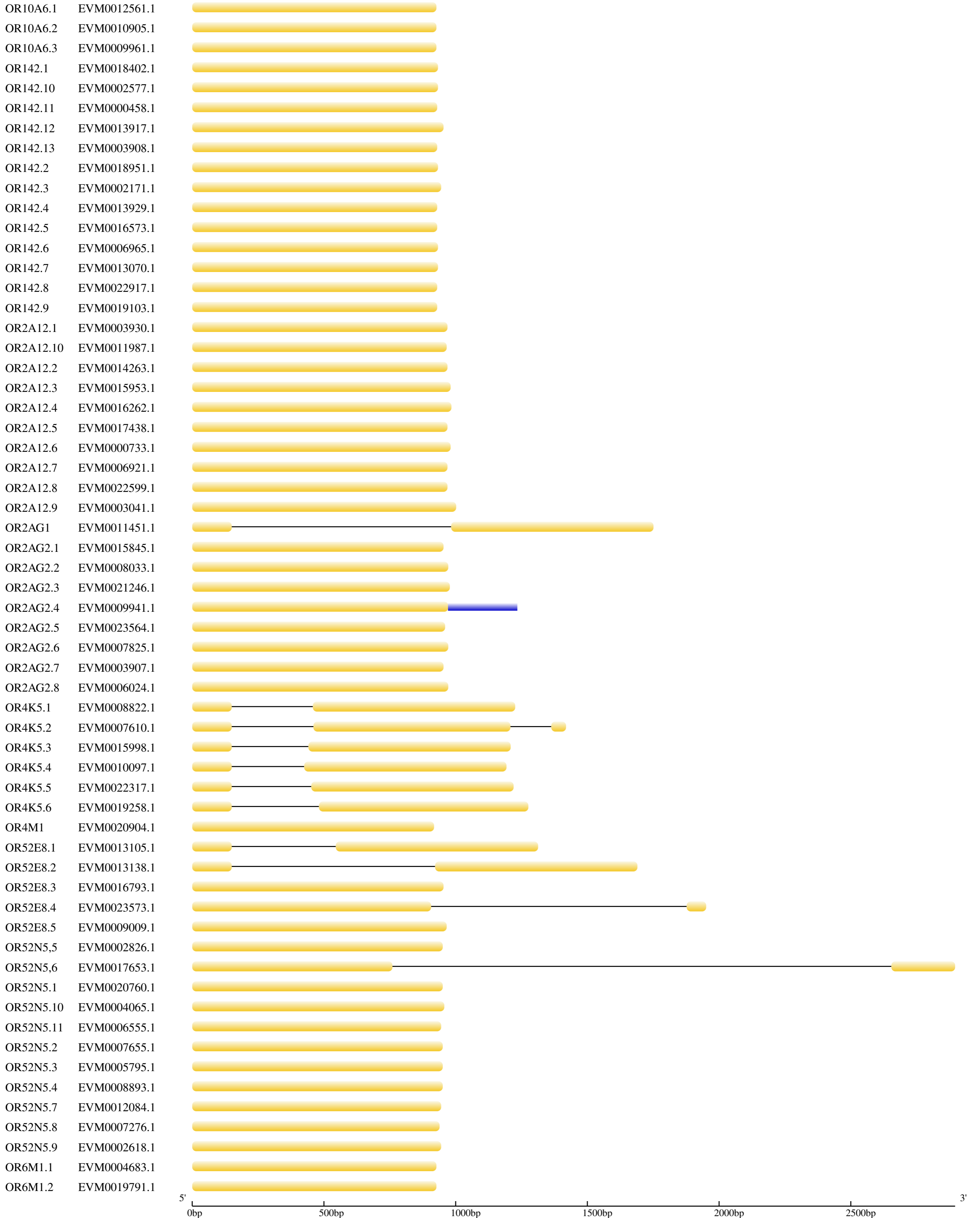

Legend:  
CDS UTR Intron

Supplement: Supplementary file 1 [file animals-13-01232-s001.zip › Figure S1.pdf]
