# Supplementary material for: Genome-Wide Identification and Characterization of Olfactory Receptor Genes in Silver Sillago (Sillago sihama)
Source: Animals (Basel). 2023 Apr 1;13(7):1232. doi: 10.3390/ani13071232 (PMC10093537; doi:10.3390/ani13071232)

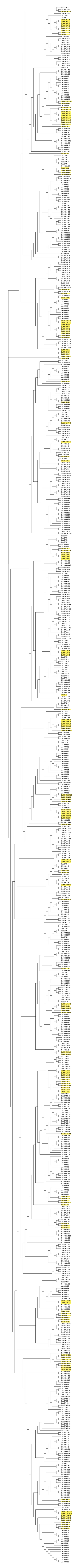

Supplement: Supplementary file 1 [file Figure S2.pdf › Figure S2.pdf]
